# Heterogeneous patterning behavior emerges from human stem cell-based embryo model

**DOI:** 10.1063/5.0329616

**Published:** 2026-11-12

**Authors:** Ian Jan, Jacob I. Evarts, Jason Y. Cain, Po-Hao Chiu, Min Yang, Neda Bagheri, Nancy L. Allbritton

**Affiliations:** 1 Department of Bioengineering, University of Washington, Seattle, Washington 98195, USA; 2 Department of Biology, University of Washington, Seattle, Washington 98195, USA; 3 Department of Chemical Engineering, University of Washington, Seattle, Washington 98195, USA; 4 Department of Obstetrics and Gynecology, University of Washington, Seattle, Washington 98195, USA; 5 Institute for Stem Cell and Regenerative Medicine, University of Washington, Seattle, Washington 98109, USA; 6 Brotman Baty Institute for Precision Medicine, Seattle, Washington 98109, USA

## Abstract

Undergoing complex self-organization, two-dimensional gastruloids derived from human pluripotent stem cells (hPSCs) are a scalable model system to study developmental processes. However, gastruloid heterogeneity has been obscured by technological limitations. A microraft-based assay was developed to screen, sort, and sequence individual living gastruloids expressing germ layer reporters to quantify the emergence of gastruloid variability. In this proof-of-concept study, distinct patterning trajectories were identified from latent representations using time-series imaging combined with self-supervised deep learning. End point gene expression of isolated single gastruloids was measured to further evaluate heterogeneity and link phenotype to gene expression. The computational analysis captured nuanced technical and biological differences among individual gastruloids across and within batches. Additionally, single gastruloid transcriptomic data revealed clusters by batch and subclusters within experimental batches. Given the small sample size, no detectable association could be inferred from the imaging and sequencing data. Orthogonal measurements will provide deeper insights from each gastruloid; therefore, the preliminary results highlight the need for optimized computational methods and adequately sized training datasets. This platform coupled both imaging and transcriptomic data for comprehensive assays of single gastruloids. Moreover, dissecting the intrinsic heterogeneity of gastruloids derived from the same parental hPSC line provides a powerful lens into the complex variability of stem cell-based embryo models. Importantly, these results call for the careful evaluation of reproducibility in human *in vitro* systems as applied to human embryogenesis, especially given the current push away from animal models.

## I. INTRODUCTION

Human embryogenesis involves the complex yet concerted organization of multiple cell types. Consequently, heterogeneity can arise among developmental processes and pregnancy outcomes. Observed as early as the first cell fate decision, heterogeneity has major implications for reproduction, as only a third of human conceptions result in live birth.^1,2^ Studying the developmental trajectories of early embryos has the potential to improve our understanding of the mechanisms of early pregnancy loss. Given technical and ethical limitations surrounding human embryos, human stem cell-based embryo models (SCBEMs) are crucial for both the fundamental understanding of healthy embryonic development and the development of reproductive treatments.^3–5^ While animal models have led to our improved understanding of embryogenesis, these models do not fully mimic the human process. For these reasons as well as ethical and cost concerns, national agencies such as the National Institutes of Health (NIH) and the U.S. Food and Drug Administration (FDA) have proposed to phase out animal models.^6,7^ An example of a human SCBEM is the two-dimensional (2D) gastruloid, termed “gastruloid” in this report. To create these entities, human pluripotent stem cell (hPSC) colonies are cultured in geometrically confined spaces coated with extracellular matrix (ECM).^8^ After cell attachment, the mature gastruloid is formed upon treatment with bone morphogenetic protein 4 (BMP4). Gastruloids display the self-patterning of an extraembryonic trophectoderm-like region and the germ layers (endoderm, mesoderm, and ectoderm) that give rise to the different organs within the embryo. This model system has been proven invaluable for both fundamental studies and for revealing clinical insights. In addition to elucidating the complex signaling cascade (consisting of the BMP, Wnt, and Nodal pathways) underlying gastrulation, gastruloids have been used to model the depletion of aneuploid cells possessing abnormal sets of chromosomes.^9,10^ Gastruloids can be cultured as scalable arrays and formed in 48 h; thus, gastruloids are an ideal embryo model to capture dynamic processes at scale via quantitative and high-dimensional measurements.^8,9,11^ Other multicellular entities—even when derived from the same cell line—exhibit significant heterogeneity across different experiments as well as within the same experiment.^12–14^ Furthermore, individual hPSCs within a single population display substantial variability, which has been hypothesized to provide hPSCs the flexibility for diverse lineage specification.^15,16^ The heterogeneity of single adherent 2D gastruloids has yet to be characterized. Currently, no high-throughput or high-content analytical technologies can quantify the emergence of heterogeneity among developing gastruloids to better understand the experimental impact of this variability.

Comprehensive characterization of the gastruloid model system calls for novel tools to connect information across multiple modalities, such as morphology and gene expression levels. Studying the complex dynamics of self-patterning during early embryonic development also requires noninvasive image-based tracking of the lineage specification within the gastruloids. The conventional approach to image analysis using human-led feature extraction can introduce bias and miss multi-dimensional features that are difficult to quantify by simple metrics.^17–19^ In response, deep learning has been used to identify cell density and SOX2 (a marker for pluripotency and the ectoderm) stability as key parameters to distinguish phenotypes—including gastrulation failure modes—of end point, fixed gastruloids.^20^ This strategy created latent representations, or embeddings, derived from intensity measurements of different cell lineage markers along the radius of each gastruloid and did not include transcriptomic data. Connecting gene expression to image features would support a deeper understanding between patterning and the underlying signaling dynamics. The recent adaptation of the microraft platform to gastruloids has opened the door to coupling high-resolution imaging followed by sorting and gene expression assay on the imaged gastruloids.^21^ Further development of the innovative microraft platform to connect time-series imaging and end point gene expression data on single gastruloids would empower detailed studies of gastruloid heterogeneity.

For the high-throughput screening and sorting of gastruloids, arrays containing hundreds of gastruloids as described previously were utilized.^21^ On these arrays, each gastruloid is positioned on an optically clear, releasable carrier or microrafts. These microrafts are uniform in dimensions with large, central, flat regions suitable for ECM placement and gastruloid formation. The microrafts possess embedded superparamagnetic beads at their edges, enabling collection by a magnetic wand upon dislodgement from the array.^21,22^ Gastruloids formed from RUES2 hPSCs on both the microraft arrays and the gold-standard flat polystyrene multi-well plates displayed clearly segregated tissue zones corresponding to three tissue layers (ectoderm, mesoderm, and trophectoderm).^21^ Gastruloids on the microrafts and polystyrene plates also closely resembled each other in overall size of the cell lineage regions. Thus, microraft arrays are an excellent culture surface for gastruloids, compatible with high-quality microscopy, and offer the potential for gastruloid sorting.

Here, we developed a novel integrated suite of tools (from imaging to mechanical sorting) to perform comprehensive assays of single gastruloids for characterizing gastruloid heterogeneity and, in the future, addressing fundamental biological questions regarding early embryogenesis. Time-series imaging across 48 h of living gastruloids developing on microrafts was performed. A single array possessed 529 microrafts, each carrying an individual gastruloid derived from cells expressing fluorescent reporters for germ layer markers. Low-dimensional latent representations were constructed from high-throughput imaging of the SOX2 reporter to identify unique gastruloid phenotypes. Directly creating the embeddings from the images requires minimal prior knowledge (thereby reducing bias), allows for fast hypothesis generation, and captures rich spatiotemporal information. The latent features enable both the quantification of variability over time and identification of potential sources of heterogeneity at the single gastruloid level. Visual inspection of latent dimensions using perturbation-based methods revealed morphological features potentially associated with key differences in self-patterning among gastruloids from multiple batches. Additionally, the same model can be trained on complete imaging time-series to produce single embeddings capturing temporal dynamics for each gastruloid. The microraft array platform also supported the isolation of single gastruloids after imaging for downstream gene expression analysis via bulk RNA sequencing (RNA-seq). No feasible approach currently can extract bulk RNA-seq data from single colonies of only ∼1000 cells while preserving imaging and other information of the colony of origin. Therefore, a commercial single-cell RNA-seq library preparation was adapted to single gastruloids for analyzing the transcriptomic variability across gastruloids between experiments and within a single experiment. Single gastruloid transcriptomics was readily performed using these combined technologies.

## II. RESULTS AND DISCUSSION

### A. Gastruloids consistently develop on microrafts

Because gastruloids derived from RUES2 hPSCs had been demonstrated previously on the microrafts, the platform was applied to a different hPSC line (RUES2 expressing the SOX2-mCitrine reporter) for the live monitoring of SOX2 dynamics.^21^ The SOX2-mCitrine reporter was employed to assess the consistency of gastruloid formation on the microrafts. Upon microscopy examination of the formed gastruloids on the microraft surface, every microraft (100%, n = 50 gastruloids) contained a gastruloid. The majority of the gastruloids (92%) were properly aligned in the centers of the microrafts (supplementary material Fig. S1). A minority of the microrafts (12%) had defects, including embedded beads that blocked parts of the gastruloid and defects in the polystyrene. Most gastruloids (94%) had properly developed ectoderms indicated by a single localized, central SOX2+ region. Only three gastruloids exhibited dispersed or irregularly shaped ectoderm regions. Additionally, 24% of the gastruloids had adjacent cell aggregates, which formed from excess cells that detached from beyond the ECM patches. These cell aggregates did not obscure the imaging or greatly affect the development of the gastruloids (only one gastruloid with poor ectoderm formation had adjacent aggregates). Previously, 90 ± 20% of the stained (dyes and/or antibodies) gastruloids were reported to be properly aligned.^21^ Gastruloid eccentricities were 0.2 ± 0.1 and 0.3 ± 0.1 for the full gastruloid and the SOX2+ region, respectively (n = 43 gastruloids). The radius of the SOX2+ region (160 ± 10 *μ*m) was smaller than that of the full gastruloid (270 ± 10 *μ*m). These values are consistent with corresponding ones previously reported for eccentricity (0.3 ± 0.0 for the full gastruloid and 0.3 ± 0.0 for the SOX2+ region) and radius [251 ± 4 *μ*m for the region of interest (ROI) and 151 ± 7 *μ*m for the SOX2+ region].^21^ These data suggested that the gastruloids derived from reporter cells were cultured with the same circular geometries and level of consistency as the gastruloids previously grown on the microrafts.

### B. Gastruloids exhibit remarkable heterogeneity

Despite the similarities between gastruloids, this model system revealed morphological heterogeneity even within the same array. To examine the variability of the SOX2 signal during self-patterning, imaging time-series (nine YX images total with 6-h intervals over 48 h) of gastruloids were captured from seven batches derived from three aliquots (A, B, and C) of the same hPSC line [Fig. 1(a) and Table I]. For each YX image slice, a 16-dimensional (16D) latent representation was generated via an autoencoder model in a self-supervised manner [Fig. 1(b)]. The embeddings were visualized by Uniform Manifold Approximation and Projection (UMAP) dimensionality reduction.^23^ Given the stochastic nature of model runs and UMAP, the robustness of both processes was verified by performing multiple independent model runs and creating UMAP plots while varying the number of neighbors parameter (supplementary material Fig. S2). The number of neighbors parameter was set to 15 to balance both the local and global structure of the latent representations. From the UMAP plot, the embeddings were grouped together at the initial time point but dispersed by the final time point [Fig. 2(a)]. There was a general trend of the temporal trajectories of the latent representations toward the same direction along the UMAP1 axis. Notably, the latent representations dispersed along both UMAP1 and UMAP2 axes over time. To describe the directional changes of the latent representations in reference to a common origin, displacement vectors were calculated by subtracting each standardized latent representation from the latent representation of the same gastruloid at the 0 h time point [Figs. S2(a) and S2(b)]. The pairwise distance between the displacement vectors at 48 h was significantly greater than that at 6 h (paired t-test, p = 0.010). This suggests that the variability of latent representations increases over time. Based on the spacing of latent representations at each time point, the morphology of the gastruloids was similar initially after seeding but deviated by the final time point at 48 h. Extracting latent features from thousands of gastruloid images across nine time points revealed temporal dynamics of SOX2 expression in the developing gastruloids.

**FIG. 1.**
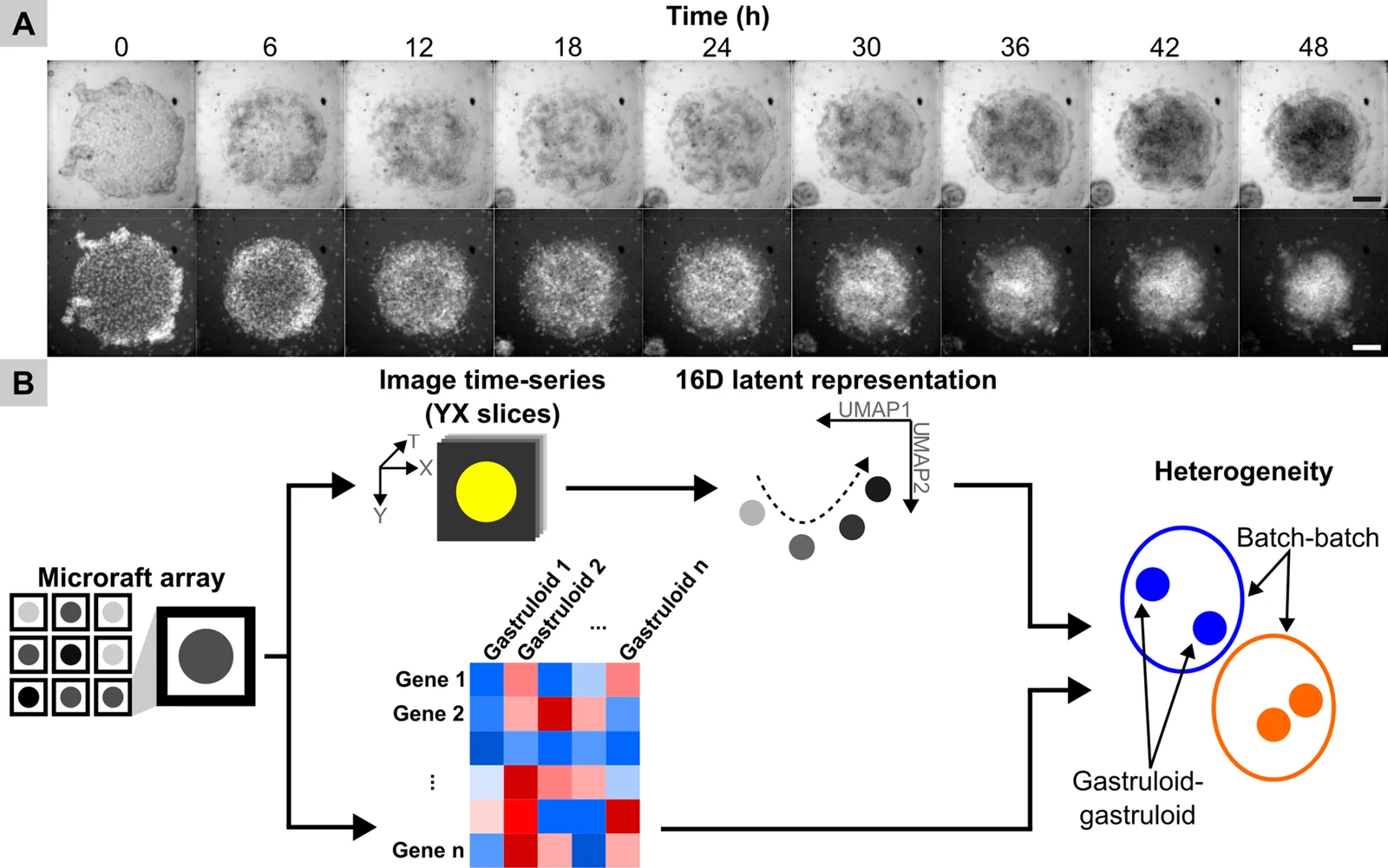
Exploring single gastruloid heterogeneity. (a) Shown are sequential bright-field (upper row) and fluorescence (lower row) images using widefield microscopy of a single microraft possessing hPSCs expressing a SOX2-mCitrine reporter. BMP4 was added to the cultures at time 0 h to initiate gastruloid formation. Scale bars, 100 *μ*m. (b) Schematic of the live time-series imaging and end point RNA-seq workflow. SOX2 dynamics and gene expression profiles of single gastruloids were acquired. 16D latent representations were formed from individual YX slices of imaging time-series (widefield microscopy) of each gastruloid and visualized via UMAP dimensionality reduction. Gastruloid heterogeneity across and within experimental batches was assessed using the image-based latent representations and sequence-based gene expression profiles.

**TABLE I.** Experimental batches with the number of subcultures (passage number) corresponding to three aliquots of a single RUES2 line.

Aliquot	Batch	Passage number
A	0609	79
A	0617	81
B	0623	77
B	0630	79
C	0715	77
C	0722	79
C	0729	81

**FIG. 2.**
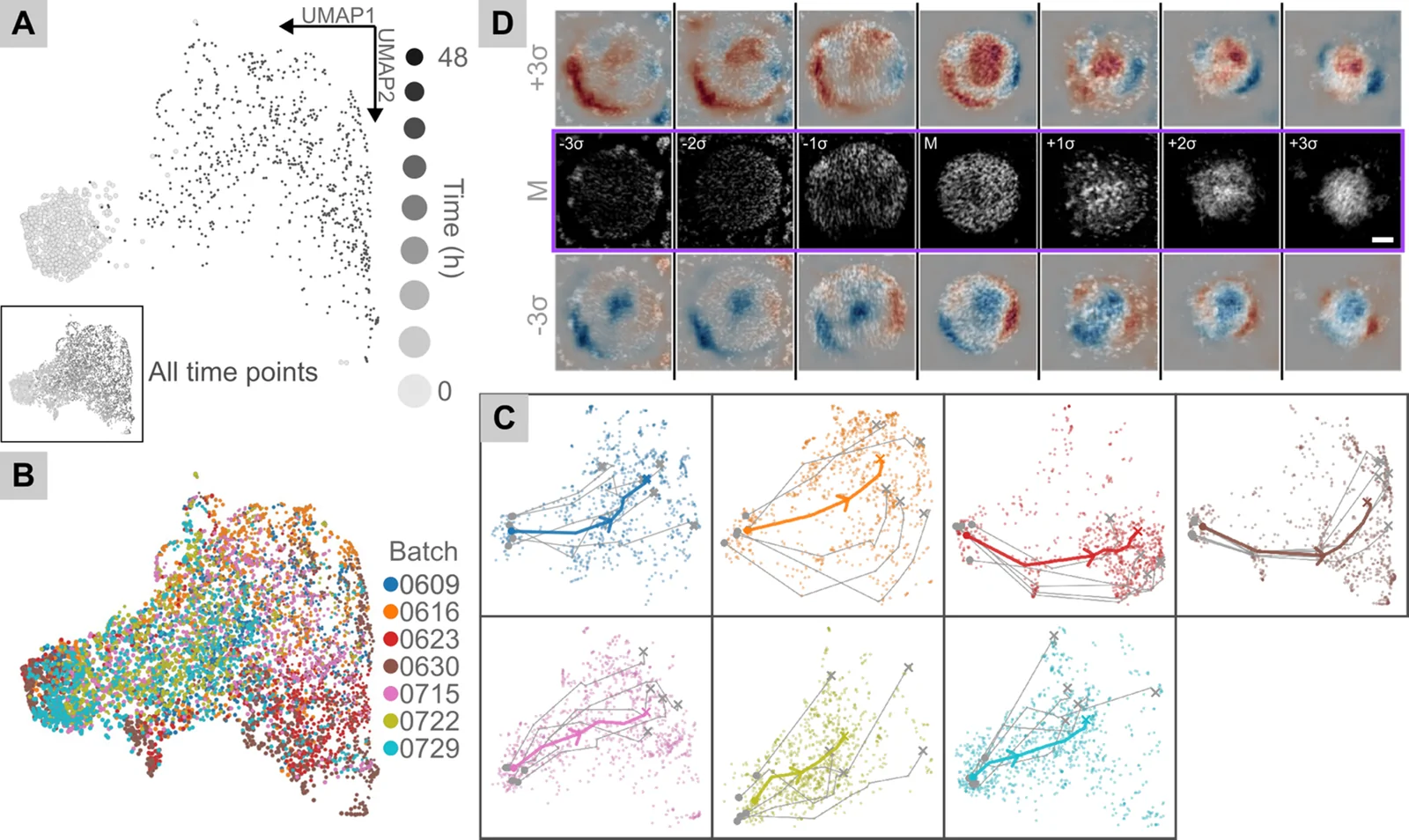
Static latent representations learned from individual gastruloid images reveal heterogeneity. (a) UMAP plots visualizing 16D latent representations learned from individual static YX slices from imaging time-series of gastruloids. The main plot shows latent representations from initial and final time points (relative sizes of markers in the legend are not drawn to the same scale as markers in the plot), while the subplot shows latent representations from every time point. Shade within the gradient corresponds to the time point (6-h interval) during the 48-h differentiation protocol. (b) UMAP plot visualizing latent representations from every time point where hue corresponds to batch. (c) UMAP plots for each batch visualizing average temporal trajectories of all batch-specific latent representations and trajectories of five randomly sampled gastruloids. The start (circle) and end points (x) for each trajectory were labeled. Hue corresponds to batch. For panels (a)–(c), N = 7 batches. (d) Latent walk of latent feature #6. Along the middle row (purple), seven example gastruloids with increasing latent feature #6 were ranked in the range of ±3 standard deviations (σ), including the median (M). For each column, visual counterfactual alteration of latent feature #6 in the range of ±3 standard deviations (σ), including the median (M), was displayed. Visual counterfactual alterations were normalized by the original example image, where red/blue indicate positive/negative changes in intensity, respectively. Scale bar, 100 *μ*m.

To understand the dispersion of latent representations in the UMAP plot, gastruloid images randomly sampled throughout the plot were visually inspected. This workflow was designed to scale for large screens of potentially millions of gastruloids; therefore, basic processing was performed on the images for illumination correction, and the subsequent latent space was visually explored. Because the latent features are expected to capture technical artifacts, including embedded beads and adjacent aggregates, these latent features can be used for subsequent automatic filtering as a strategy to distinguish technically distinct gastruloids. Early-stage gastruloids (mainly represented by scattered dim SOX2 signal) were shifted toward higher UMAP1 values (supplementary material Fig. S3). On the other hand, late-stage gastruloids (either exhibiting dense central or dispersed SOX2 regions) were shifted toward lower UMAP1 values. Middle-stage developing gastruloids exhibited SOX2 regions more concentrated than those of early-stage gastruloids but less dense than those of late-stage gastruloids. There were outliers, such as early-stage gastruloids with concentrated SOX2 signal around the gastruloid edge. The experimental batch associated with each sampled latent representation and corresponding image was labeled, revealing latent representations from multiple batches were scattered throughout the plot. Interestingly, latent representations of uncentered gastruloids derived from a single batch were localized in the bottom right corner of the plot. Gastruloids are known to undergo complex multicellular processes and thereby reveal morphological differences throughout their development.^8,9,11^ Both the developmental stage of the gastruloid and technical artifacts within the images appear to drive the remarkable heterogeneity of the latent representations.

### C. Multiple factors support gastruloid variability

The experimental batch has been shown as a clear source of variability in other multicellular model systems;^12–14^ therefore, the role of batch in gastruloid heterogeneity was investigated. To observe batch–batch variability, the UMAP visualization of the latent space was dissected by batch [Figs. 2(b) and 2(c)]. The UMAP plot of the static latent representations revealed the scattering of latent representations associated with the different batches [Fig. 2(b)]. Importantly, the latent representations at each time point for a single gastruloid can be connected to trace a temporal trajectory describing the SOX2 dynamics during the gastruloid development. Separated by individual batches, the UMAP subplots displaying the average temporal trajectory exhibited clear batch effects [Fig. 2(c)]. The subplots for batches 0623 and 0630 showed latent representations that were concentrated in the bottom right corner (low UMAP1 and high UMAP2 values). Dissecting the composite UMAP plot with all batches suggested that cell culture aliquot (A, B, and C) and passage number (77, 79, and 81) may also contribute to gastruloid variability (supplementary material Fig. S4). Specifically, both batches 0623 and 0630 were the only batches derived from aliquot B, which uniquely produced latent representations localized in the bottom right corner. Regardless, the average trajectories follow a general trend toward lower UMAP1 values [Fig. 2(c)]. Although the gastruloids were derived from cells of multiple thawed aliquots of a single cell line, the experimental batch introduced variability throughout the self-patterning. In addition to capturing the heterogeneity of the SOX2 signal at each time point, the latent representations assist in describing the complex temporal changes of single gastruloids between batches.

Pooled analyses of single gastruloids can easily overlook the heterogeneous dynamics at the single gastruloid level. Consequently, the trajectories of individual gastruloids were randomly sampled for each batch [Fig. 2(c)]. The sampled trajectories started in similar areas of the subplots and followed the general trend of the batch-specific averaged trajectory. However, the sampled trajectories generally deviated within the first 24 h and ended in diverse regions. Some trajectories started and ended in similar places (as shown in batches 0623 and 0630), while others started in similar regions before ending in different areas (as shown in batches 0609 and 0729). For some batches, the sampled trajectories appeared to even follow different sets of paths. The sample trajectories sometimes followed similar paths as the corresponding batch-specific averaged trajectory (as shown in batches 0630 and 0715). On the other hand, some sampled trajectories followed paths above and below the corresponding batch-specific averaged trajectory (as shown in batches 0616 and 0729). Similar observations were made from randomly sampled trajectories from the subplots corresponding to cell culture aliquots and passage numbers (supplementary material Fig. S4). In these subplots, the starting and ending points as well as the trajectory paths all varied. The trajectories from single gastruloids revealed remarkable heterogeneity of SOX2 dynamics that were masked by the batch-level averaged trajectories [Fig. 2(c)]. Previously, high-throughput screening of single end point gastruloids showed variability across all samples despite the measured features having similar replicate-level means.^21^ Overall, the trajectories of the latent representations averaged at the batch level and randomly sampled from single gastruloids captured the emergence of morphological heterogeneity not only between but also within batches.

### D. Biologically relevant features can be extracted from gastruloid latent representations

The interpretability of the latent features extracted by deep learning approaches can be challenging, but understanding these features can uncover structures with potential biological relevance in the image data. For each latent dimension, counterfactual analyses were performed by generating a series of seven sample gastruloid images ranked by the specific latent feature within ±3 standard deviations (supplementary material Fig. S5). Each image series was visually inspected to determine the possible image features that the latent dimensions were capturing. Most of the features were not readily interpretable. However, a few latent features readily appear to be biologically relevant. Latent feature #1 appeared to encode the position of the gastruloids along the x axis of the image. Additionally, changes to latent feature #6 appeared to encode the size of the central SOX2+ region within the gastruloids of each image. Given the complex dataset, which included tens of thousands of gastruloid images across multiple time points and experimental batches, the model also likely captured abstract features. Latent representations can encompass subtle differences between images, such as small changes in 16-bit depth intensity values or abstract structures formed by the SOX2 signal, that are not obvious to the human eye. Regardless, formed from the imaging time-series of the gastruloids, the latent representations can capture nuanced but important differences with potential biological relevance between each image.

These series of real gastruloid images did not hold any of the other features constant, thereby complicating straightforward interpretation of the latent features. To address this, synthetic gastruloid images were generated from the original real images by perturbing each specific feature by ±3 standard deviations (supplementary material Fig. S6). Latent feature #6 appeared to encode a morphological feature readily; thus, visual interpretation of this latent feature was performed as a proof-of-principle. Since the changes in intensity of the generated images were not easily discernable, the synthetic images were normalized by the original real image to create a heatmap [Fig. 2(d)]. Increases/decreases in intensity are indicated by red/blue, respectively. The synthetic heatmap pairs for each image showed that SOX2 intensity mainly in the gastruloid center correlated with the value of latent feature #6. Less obvious were changes in SOX2 intensity at the colony edges. SOX2 intensity increased at the bottom left edge of the gastruloid but decreased along the rest of the edge when latent feature #6 was increased. This counterfactual analysis further supported that latent feature #6 likely encoded the radial density of the SOX2 signal. The size of the central SOX2+ region is likely biologically relevant for self-patterning. Both cell density and SOX2 stability have been identified as driving parameters for patterning variability.^20^ Interpretable features were not readily identified from the other latent features, and it is likely that abstract features may have played a role. The latent walk performed on latent feature #6 suggests that the other latent features may also capture biologically meaningful physical features. This proof-of-principle approach using both real and synthetic counterfactual images was employed to interpret abstract latent features with minimal prior knowledge.

### E. Latent representations constructed from complete imaging time-series capture temporal dynamics

Because embryogenesis is a dynamic process rich with temporal information, the model was applied to study the entire sequence of each gastruloid evolving over time. The goal was to gain more insights into the heterogeneous progression of self-patterning among gastruloids from different experimental batches. Specifically, the model was trained on a series of YX images captured over time, which form image stacks with three dimensions (3D) (TYX). The model constructed a single 16D latent representation that encapsulated the SOX2 patterning over the entire time-series for each gastruloid [Fig. 3(a)]. UMAP dimensionality reduction was performed on the dynamic latent representations to show clear batch effects [Fig. 3(b)]. As a case study for qualitative interpretation via counterfactuals, latent feature #5 appeared to encode the rate of condensation of the SOX2+ region at the gastruloid center [Fig. 3(c)]. The SOX2 signal is less localized at the gastruloid center at higher than lower values of latent feature #5. Interestingly, the example gastruloids had similar morphology at the initial time point but showed clear differences by 24 h. These dynamic latent representations encompassing the entire time-series captured temporal information missing from the static latent representations formed from YX slices at single time points. The static latent representations required connecting the embeddings from each time point for each gastruloid. Both types of latent representations provide important insights into the SOX2 dynamics across the different batches. In the UMAP plot of the dynamic embeddings, the latent representations from batches 0623 and 0630 (aliquot B) are more similar to each other than those from batches 0722 and 0729 (aliquot C) [Fig. 3(b)]. The average trajectories corresponding to these batches in the UMAP plot of the static latent representations support this observation [Fig. 2(c)]. Taken together, the latent representations extracted complex features from high-dimensional imaging data to describe the time-dependent emergence of gastruloid heterogeneity.

**FIG. 3.**
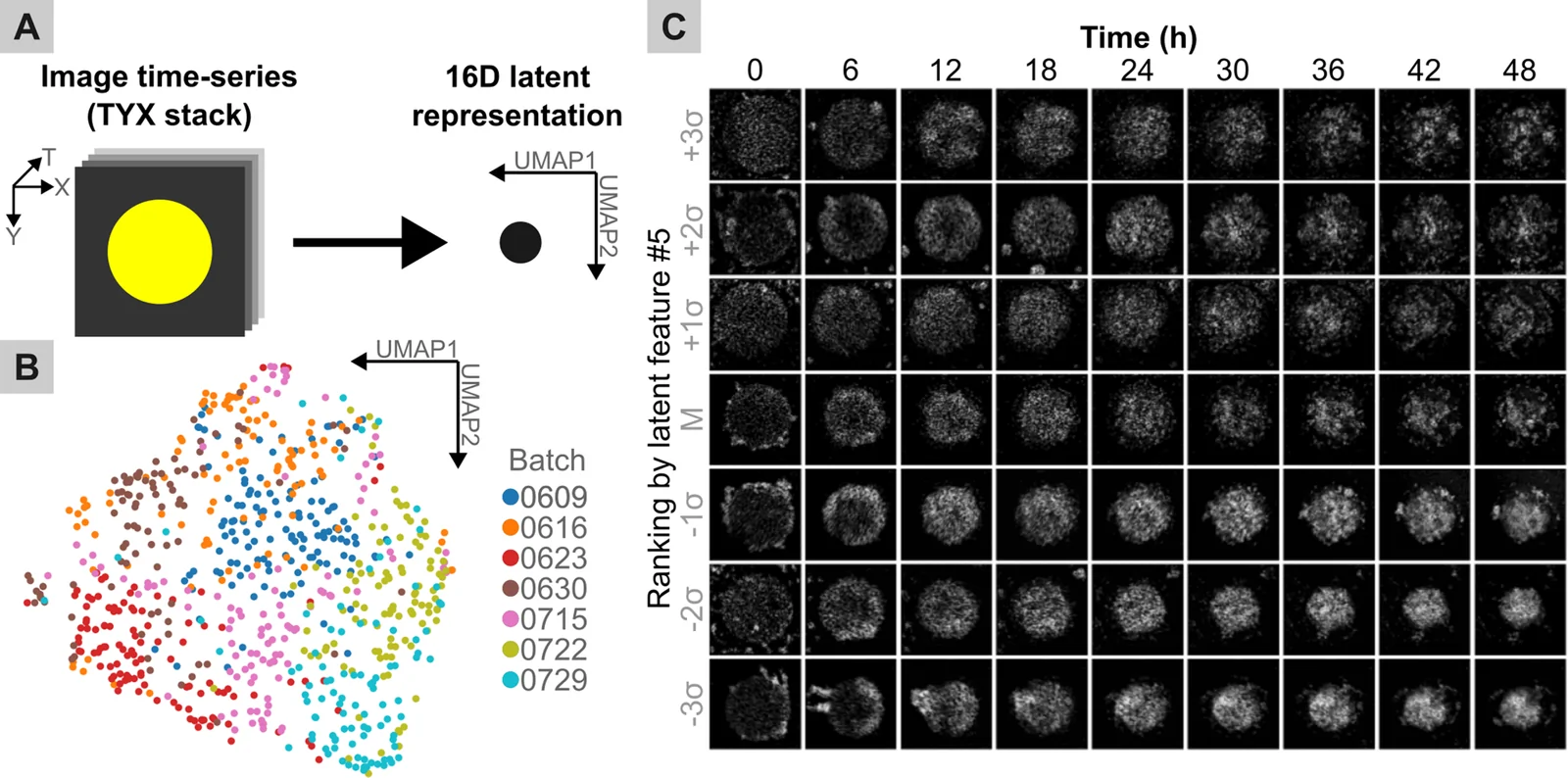
Dynamic latent representations learned from complete gastruloid imaging time-series. (a) Schematic of workflow where each 16D latent representation was formed from a dynamic TYX stack of complete imaging time-series of a single gastruloid. (b) UMAP plot visualizing latent representations learned from 3D imaging time-series stacks. Hue corresponds to batch. N = 7 batches. (c) Latent walks for latent feature #5 displaying imaging time-series over a 48-h differentiation protocol of seven example gastruloids ranked by latent feature #5 in the range of ±3 standard deviations (σ), including the median (M).

### F. Transcriptomic profiles of single gastruloid reveal heterogeneity

For a comprehensive assessment of gastruloid variability, the gene expression profiles of single gastruloids were investigated. A total of 29 gastruloids from 2 batches (n_0722_ = 13 gastruloids, n_0729_ = 16 gastruloids) were isolated, sequenced, and analyzed. Principal component analysis (PCA) was performed for dimensionality reduction of the gene expression data to visualize the sample variance of the data clearly [Fig. 4(a)]. The gene expression profiles were clearly separated by batch. Additionally, the top 1000 highly variable genes were visualized with a heatmap, where clustering was performed on both the genes and samples [Fig. 4(b)]. The samples were not only clustered by batch but also subclustered within each batch. Out of the 1000 genes, the top 10 highly variable genes were labeled. Most of the top 10 highly variable genes play critical roles in embryogenesis (Table II). For instance, CER1 and DKK4 are antagonists in the BMP, Nodal, and Wnt signaling cascade.^24,25^ Other genes are involved in the patterning of specific structures or tissues, such as FGF17 (mesoderm and midbrain–hindbrain boundary), CYP26A1 (rostral neural plate and embryonic tail bud), and MIXL1 (mesoderm and endoderm).^26–29^ The gastruloids appear to exhibit single-gastruloid variability across the different batches on both phenotypic and transcriptomic levels. This feasibility study serves as the basis for future large-scale screens with additional independent batches. Moreover, targeted studies of the highly variable genes can be performed to determine whether the heterogeneity is derived from extrinsic or intrinsic factors.

**FIG. 4.**
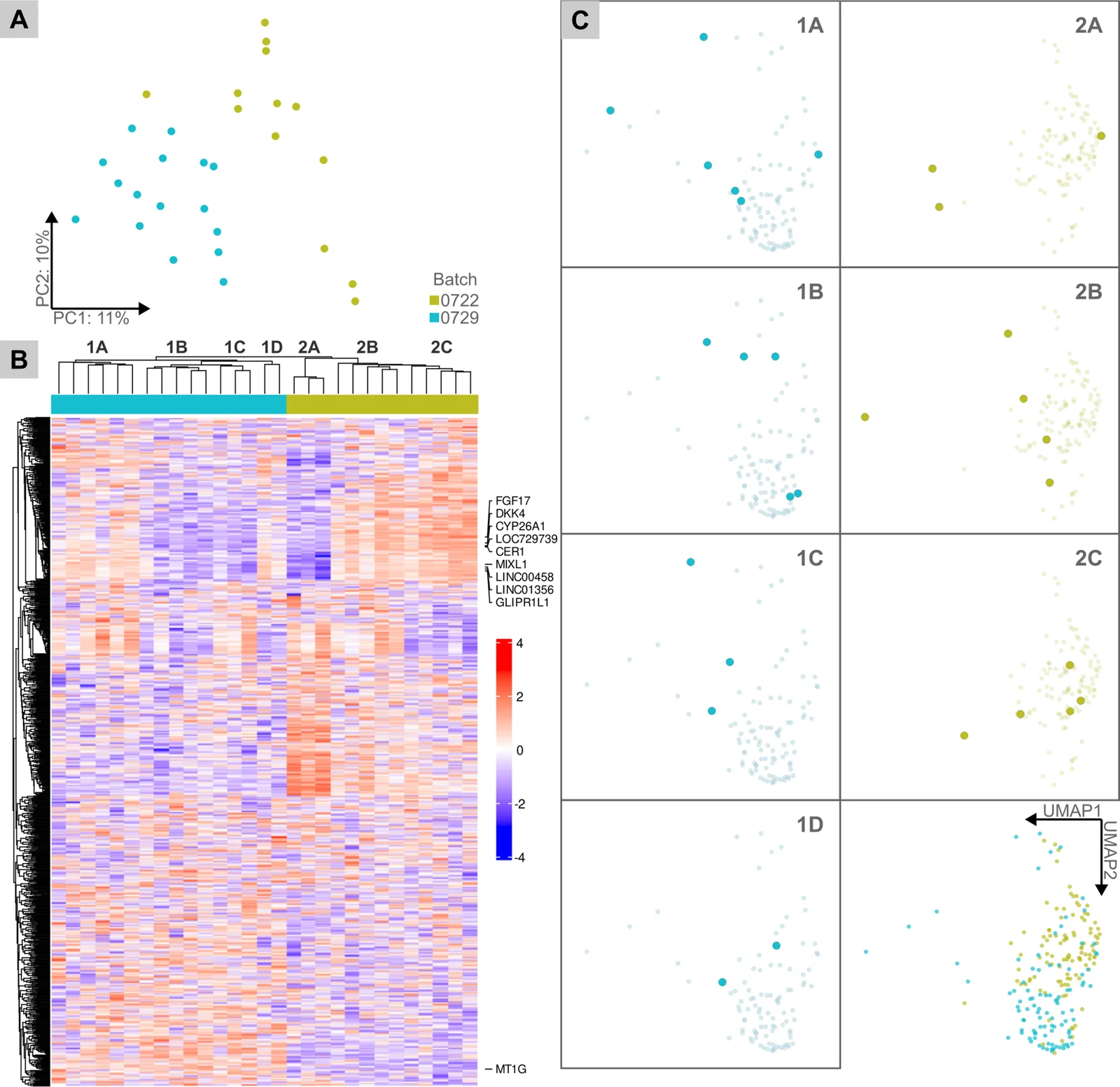
Transcriptomic profiles of single gastruloids are heterogeneous. (a) Principal component analysis (PCA) plot of gene expression profiles from single gastruloids. Proportions of variances indicated for PC1 and PC2. Hue corresponds to batch. (b) Heatmap displaying normalized relative expression of the top 1000 highly variable genes. Hierarchical clustering of both genes and samples (hue corresponds to batch) was performed using Pearson correlation distance with average linkage. Sample clusters and top 10 highly variable genes were labeled. (c) UMAP plots visualizing the dynamic latent representations of the sequenced gastruloids (large markers) in each subcluster and of the gastruloids in each batch (small markers). Hue corresponds to batch. For panels (a)–(c), N = 2 batches, n_0722_ = 13 gastruloids, and n_0729_ = 16 gastruloids were sequenced.

**TABLE II.** Top 10 highly variable genes expressed by single gastruloids.

Rank	Gene	Associated processes	References
1	CER1 (cerberus 1)	Multifunctional inhibitor of BMP, Nodal, and Wnt signaling.	24
2	DKK4 (dickkopf WNT signaling pathway inhibitor 4)	Wnt/β-catenin antagonist.	25
3	FGF17 (fibroblast growth factor 17)	Involved in mesoderm differentiation and in establishing the midbrain–hindbrain boundary.	26, 27, 30
4	CYP26A1 (cytochrome P450 family 26 subfamily A member 1)	Helps regulate retinoic acid (RA), which is critical in vertebrate embryo patterning and organogenesis. Murine CYP26A1-null mutants died during mid-late gestation and displayed morphogenetic defects in the rostral neural plate and embryonic tail bud.	28
5	GLIPR1L1 (glioma pathogenesis-related protein 1)	Part of mammalian cysteine-rich secretory proteins, antigen 5, and pathogenesis-related 1 proteins (CAP) superfamily. Facilitates sperm–oocyte interaction.	31, 32
6	MIXL1 (mix-like 1)	Crucial for mesodermal and endoderm development. Mouse epiblast stem cells showed temporal differences in MIXL expression, where cells with delayed MIXL upregulation were less responsive to NODAL signaling.	29, 33, 34
7	LINC00458 (long intergenic non-protein coding RNA 458)	Elevated in response to substrate stiffness. Promotes endoderm specification via SMAD2/3.	35
8	LINC01356 (long intergenic non-protein coding RNA 1356)	Elevated in response to substrate stiffness. Unclear role.	35
9	LOC729739	Peptidylprolyl isomerase A (cyclophilin A) pseudogene.	
10	MT1G (metallothionein 1G)	Part of a group (metallothionein or MT) of genes encoding metal-binding proteins that perform a variety of roles (including metal homeostasis, oxidative stress, cell proliferation and differentiation, and inflammation). Interestingly, higher expression of MT1G was associated with preterm labor in fetal membranes and myometrium.	36, 37

### G. Multimodal characterization of single gastruloids connects phenotypic and transcriptomic information

Whether the latent representations and gene expression profiles provide correlated or orthogonal information about single gastruloids was unknown. The sorted microrafts have the powerful ability to connect imaging and sequencing data for the same biological sample. Therefore, the dynamic latent representations (encapsulating the complete imaging time-series) of the sequenced gastruloids from batches 0722 and 0729 were initially visually assessed by subclusters (according to the gene expression profiles) [Fig. 4(c)]. The gene expression for the gastruloids from batch 0722 revealed three subclusters, while those for gastruloids from batch 0729 showed four subclusters. In general, the latent representations of gastruloids within the same subcluster did not appear to be closely located in the UMAP subplots derived from the imaging data. The embeddings for subcluster 1B (batch 0729) were dispersed along the imaging UMAP2 axis, while those for subcluster 2B (subcluster 0722) were spread out along the imaging UMAP1 axis. Consequently, the relationship of the different data modalities required further analysis.

To better identify a possible correlation between the imaging latent dimensions and the gene expression levels of single gastruloids, the normalized values of the imaging latent dimensions and gene expression levels of the top 10 highly variable genes were compared via heatmaps (supplementary material Fig. S7). The signed similarity metric (dependent on both sign and magnitude) was calculated for each latent dimension and gene expression level pair. In general, there were no clear consistent patterns across the heatmaps. The similarity metrics of the samples within the same sequencing subclusters did not show trends. Because single genes can perform multiple functions, the connection between the imaging latent dimensions and gene expression levels was likely too complex for this simple visual examination. Notably, the microraft technology can enable these challenging analyses by integrating both imaging and sequencing data for single gastruloids.

## III. CONCLUSIONS

Gastruloids serve as a scalable model to quantitatively recapitulate aspects of early lineage specification in human embryogenesis; however, analytical techniques for assessing the emergence of heterogeneity in individual gastruloids derived from thawed aliquots of a single cell line have yet to be developed. The automated microraft array platform empowered the first comprehensive single gastruloid assay by coupling time-series imaging of a fluorescently tagged lineage (SOX2) reporter in living gastruloids and end point transcriptomic data from individual gastruloids. Consequently, the results from this study suggest key heterogeneous patterning behavior and gene expression profiles among gastruloids between and within batches. Unique advantages of this technology include (i) a novel computational pipeline that extracts embeddings directly from the imaging time-series of the SOX reporter to generate hypotheses regarding biological variability and (ii) the ability to connect RNA-seq data to individual imaged gastruloids. Observing both morphological and transcriptomic information simultaneously allows the parsing of single gastruloid heterogeneity within the same batch, which would be overlooked with bulk or pooled analyses.

The proof-of-principle demonstration of this new workflow revealed exciting future directions for large-scale screens of single gastruloids. No detectable association could be inferred from the imaging and transcriptomic data from a small set of gastruloids (n = 29 gastruloids) from two batches. Consequently, if the modalities are orthogonal measurements, integrating both modalities for each gastruloid can potentially provide rich information about unique patterning behaviors. Imaging can also be used for the automatic filtering of distinct gastruloids with technical artifacts and/or unique biological features for sequencing. These preliminary findings warrant larger-scale experiments and optimized computational approaches to determine the connection between multiple data modalities for quantifying biological heterogeneity in gastruloids. Multimodal characterization is likely required to elucidate novel combinatorial relationships between the imaging and sequencing data. To better identify the relationship between the imaging latent representations and gene expression profiles, the dimensionality reduction method could be optimized. UMAP dimensionality reduction is a non-linear approach to preserve the local and global structure of multi-dimensional data.^23^ This abstraction can result in imaging latent representations of gastruloids with similar gene expression to appear unclustered. Alternatively, a computational pipeline that integrates both imaging and sequencing data could be developed. Recent deep learning-based approaches have been designed to incorporate imaging modalities for multi-omics analysis.^38–40^ Acquiring high-resolution and high-content imaging of the gastruloids could address the limitations of the widefield microscope used here when detecting the low signal from the live reporters. Because only a single SOX2 reporter and RUES2-derived cell line were studied, incorporating additional lineage markers and independent hPSC lines can strengthen the biological generalizability of gastruloid heterogeneity.

The variability of the embryonic model system suggested by this technical study has important implications in biomedical research. Human models may provide improvements in translatability, reproducibility, and cost-effectiveness over their animal counterparts; however, the gastruloid heterogeneity derived from homogeneous culture conditions calls for careful experimental design that considers single gastruloid differences. Although the variability of *in vitro* models is often reduced for consistency in applications such as drug screens, technical noise of these models can instead be exploited as a proxy for biological noise of diverse developmental dynamics.

## IV. METHODS

### A. Microraft array fabrication

Microraft arrays formed on a polydimethylsiloxane (PDMS) (#2065622, Dow Silicones Corp., Midland, MI) template possessed 529 polystyrene microrafts (789 × 789 × 100 *μ*m^3^, L × W × H dimensions) and were fabricated using methods previously described.^21^

### B. Cell culture and gastruloid formation

RUES2 human pluripotent stem cells (hPSCs) (NIHhESC-09-0013) expressing a SOX2-mCitrine reporter were cultured in mTeSR Plus medium (#100-0276, STEMCELL Technologies, Vancouver, Canada) at 37 °C and 5% CO_2_ on multi-well plates coated with 1% Matrigel (#354234, Corning Inc., Corning, NY).^41^ The cells were detached for subculture using Accutase (#07920, STEMCELL Technologies, Vancouver, Canada) and replated in mTeSR Plus containing 10 *μ*M Rho-associated kinase (ROCK) inhibitor Y-27632 (#B1293, APExBIO Technology LLC, Houston, TX) for 24 h.

Gastruloids were formed on micropatterned microraft arrays as previously described.^21^ Briefly, poly(l-lysin)100-grafted-poly(ethylene glycol)114-methoxy (PLL-g-PEG) (100 repeating units, 5000 Da, 20%; #11354, Nanosoft Polymers, Winston-Salem, NC) was diluted in PBS (#46-013-CM, Corning Inc., Corning, NY) and grafted onto the surface of a microraft array at 4 °C overnight. Circular patterns centered on each microraft and free of PLL-g-PEG were formed via photoablation by illuminating with UV light through a photomask. The circular regions were then backfilled by incubation with 1% Matrigel (#354234, Corning Inc., Corning, NY) diluted in Advanced Dulbecco's Modified Eagle Medium (DMEM)/F12 (#12634-010, Gibco, Waltham, MA) media supplemented with 0.1 mg/ml gentamicin sulfate (#30-005-CR, Corning Inc., Corning, NY) at 4 °C overnight and 25 °C for 1 h before use. hPSCs were incubated with 10 *μ*M Y-27632 for 1 h, washed with PBS, incubated in Accutase at 25 °C for 3 min, washed with DMEM/F12, and centrifuged at 200 g for 3 min. The cells were resuspended, and 1.8 × 10^6^ cells were seeded in mTeSR Plus with 10 *μ*M Y-27632 on the array. The cells were allowed to attach onto the array at 37 °C and 5% CO_2_ for 2 h. Subsequently, the array was placed into mTeSR Plus containing 50 ng/mL bone morphogenetic protein 4 (BMP4) (#314-BP-010, R&D Systems, Minneapolis, MN), and the micropatterned cells were cultured at 37 °C and 5% CO_2_ for 48 h.

Three frozen aliquots (A, B, and C) of the same RUES2 cell line subculture were thawed to form gastruloids on the micropatterned microrafts (Table I). Each experimental batch was defined as a technical replicate initiated on a single day. The number of subcultures, or passages, was limited to five for each thawed aliquot.

### C. Gastruloid imaging and sorting

The automated microscopy system described previously was used to acquire both transmitted light and fluorescence images of each microraft array.^21^ Custom MATLAB (MathWorks, Natick, MA) code utilizing a Micro-Manager (Open Imaging, San Francisco, CA) core controlled the system. The platform consisted of the following components: an inverted MVX10 MacroView fluorescence microscope (Evident Scientific, Tokyo, Japan) equipped with a 2x wide-field objective (0.25 NA); a motorized H112A microscope stage (Prior Scientific Inc., Rockland, MA); an ORCA-Flash4.0 V.3 digital CMOS camera (Hamamatsu Corp., Bridgewater, NJ); a Lumen 200 mercury lamphouse (Prior Scientific Inc., Rockland, MA); a LB10-NWIQ filter wheel with SmartShutter and an emission LB10-NWE filter wheel (Sutter Instrument, Novato, CA) fitted with an 8907 ET—ECFP/EYFP/DsRed filter set (Chroma Technology Corp, Bellow Falls, VT). Time-series imaging of the microrafts was performed over 48 h at 6-h intervals using the 4× magnification (1.7 *μ*m/pixel). During this time, the arrays were housed within an incubation chamber maintaining 37 °C, 5% CO_2_, and 55% humidity levels.

Single gastruloids were isolated with the automated sorting system previously described.^21^ Microrafts were released via an automated release device consisting of a thin needle and transferred using a microwand collection assembly from the array into individual wells of non-binding U-bottom 96-well plates (#650901, Greiner Bio-One, Monroe, NC). During microraft collection, each plate well contained mTeSR1 (#85857, STEMCELL Technologies, Vancouver, Canada). The medium was removed after microraft collection and replaced with 5 *μ*l RNase-free 1X PBS (#BM-220S-DR, Boston BioProducts, Milford, MA) on ice.

### D. Feature measurement from end point gastruloids

The eccentricity and radius were measured from manually drawn masks for the region of interest (ROI) surrounding each gastruloid and the SOX2+ region of each end point gastruloid. Only gastruloids with properly formed ectoderms and not contacting the border were analyzed.

### E. Image feature model training

To analyze the morphological variance over time, an autoencoder framework with a single encoder and two decoders was designed. This architecture processed individual images independently while extracting meaningful spatial (morphological) features with a temporal context. To mitigate uneven background illumination and improve contrast, the background of each image was removed by performing Gaussian blur (σ** **=** **50) and subtracting the blurred image from the original image. The processed images were then normalized to train the model. The encoder consisted of a convolutional neural network (CNN), mapping an input image to a low-dimensional latent representation of 16 dimensions (16D). Subsequently, this vector was passed into the two decoders: a spatial decoder for image reconstruction and a temporal decoder for predicting the relative time point. The encoder comprised a series of convolutional blocks and max-pooling layers. The spatial decoder (architectural mirror of the encoder) used convolutional and upsampling layers to reconstruct the image. The temporal decoder was a shallow multi-layer perceptron (MLP). To balance model efficacy and parsimony, the latent space was set to 16D (supplementary material Fig. S8). Larger dimensions introduced diminishing returns in model performance with regard to the loss function.

Model training was achieved by minimizing the weighted sum of the two objective functions (one for each decoder) defined as the total loss, *L_total_*

$$L_{\text{total}}=\alpha L_{\text{spatial}}+\left(1-\alpha \right)L_{\text{time}}.$$ (1)

In Eq. (1), *L_spatial_* is the pixel-wise mean squared error (MSE) used for optimizing image reconstruction, and *L_time_* is the cross-entropy loss applied to the discrete time point prediction. The hyperparameter *α* defines the prioritization of the latent representations encoding spatial or temporal information (supplementary material Fig. S9). Applying more weight to the temporal decoder loss improves the interpretability of the trajectories of the latent representations. The models were optimized using the Adam optimizer.^42^ Key hyperparameters (including optimizer hyperparameters, *α*, and dimensionality) were determined through a grid search aiming to minimize the combined spatiotemporal loss on a dedicated validation set. All complete imaging time-series (where an image of the microraft was detected and segmented at each time point) were included in the dataset. The dataset was partitioned into training (60%), validation (20%), and test (20%) sets, ensuring images from the same gastruloid remained within one set to prevent data leakage. Training procedures were performed using the PyTorch deep learning framework version 2.5.1.^43^ Imaging latent representations from only the test subset were visualized in the dimensionality reduction plots.

### F. Bulk RNA-seq

Cell pellets stored at −80 °C were thawed, centrifuged briefly to collect contents, suspended in an additional 7 *μ*l of ice-cold 1X PBS, and mixed by pipetting several times. Subsequently, 10 *μ*l of each sample were transferred to wells on a 384-well source plate (#264574, Thermo Fisher Scientific, Waltham, MA). The following steps used reagents supplied with the SMART-Seq Pro Application Kit—2 Chip (#640257, Takara Bio USA, San Jose, CA). First, a 6X dispense master mix containing 2.4 U/*μ*l RNase Inhibitor, 0.06% BSA, and 6X Second Diluent was prepared, and 2 *μ*l of this was added to each well on the 384-well source plate and mixed by pipetting. All samples were then immediately dispensed into a 5184-nanowell 350v Chip using the iCELL8 cx Single-Cell System such that each sample was dispensed into 12 replicate wells on the nanowell chip. All subsequent steps were conducted according to the manufacturer's instructions for the SMART-Seq Pro Application Kit. After extraction from the chip, libraries were quantified using the KAPA Library Quantification Kit for Illumina (#KK4824, Kapa Biosystems, Wilmington, MA). Library size was confirmed using a 4200 TapeStation (Agilent, Santa Clara, CA). The library was then sequenced on one lane of a NovaSeq X Plus 10B flow cell (#20085594, Illumina, San Diego, CA) using a paired-end 150 base-pair sequencing configuration. Median sequencing yield was 22.8 M read pairs (range 0.5–60.7 M).

Sequencing reads were aligned to the GRCh38.p14 human genome reference assembly (GCA_000001405.29), and per-sample gene expression counts were quantified using the STAR v2.7.11b aligner. Gene expression analysis was performed via the web application, iDEP (v2.4.2, South Dakota State University, Brookings, SD).^44^ Raw gene counts were filtered for genes with counts per million (CPM) ≥0.5 in at least one sample and transformed to log-CPM values in edgeR using the log_2_(CPM + c) function, where pseudo-count c = 4. Missing values were calculated using the gene-wise median. For the heatmap visualizing highly variable genes, the processed gene counts were normalized by dividing the counts by the standard deviation for each gene.

### G. Latent dimension and gene expression level correlation

The similarity metric between each imaging latent dimension (*m*) and gene expression level (*n*) (both independently normalized between [−1, +1]) was calculated by

$$\text{Similarity}=\left\{\begin{array}{cc} -\left(1-\left|\left|m\right|-\left|n\right|\right|\right), & \text{if }mn<0,\\ \left(1-\left|\left|m\right|-\left|n\right|\right|\right), & \text{if }mn\geq 0. \end{array}\right.$$ (2)

The similarity metric was bounded by [−1, 1].

## SUPPLEMENTARY MATERIAL

See the supplementary material for the figures and corresponding descriptions.

## Data Availability

The data that support the findings of this study are openly available in Zenodo at https://doi.org/10.5281/zenodo.17073719, Ref. 45 and Dryad at https://doi.org/10.5061/dryad.1vhhmgr80, Ref. 46.
